# The Associations between Kidney Function and Sexual Dysfunction among Males and Females with Type 2 Diabetes Mellitus

**DOI:** 10.3390/medicina59050969

**Published:** 2023-05-17

**Authors:** Alexandra Katsimardou, Dimitrios Patoulias, Ioanna Zografou, Zoi Tegou, Konstantinos Imprialos, Konstantinos Stavropoulos, Maria Toumpourleka, Asterios Karagiannis, Konstantinos Petidis, Michael Doumas

**Affiliations:** 1 2nd Propedeutic Department of Internal Medicine, Aristotle University of Thessaloniki, General Hospital “Hippokration”, 54642 Thessaloniki, Greece; dipatoulias@gmail.com (D.P.); ioannazo@yahoo.gr (I.Z.); zoitegou89@gmail.com (Z.T.); kostasimprialos@hotmail.com (K.I.); konvstavropoulos@hotmail.com (K.S.); astkar@auth.gr (A.K.); athanas99@hotmail.com (K.P.); michalisdoumas@yahoo.co.uk (M.D.); 2 3rd Department of Cardiology, Aristotle University of Thessaloniki, General Hospital “Hippokration”, 54642 Thessaloniki, Greece; mtoumpourleka@gmail.com; 3 Veterans Affairs Medical Center, George Washington University, Washington, DC 20422, USA

**Keywords:** diabetic kidney disease, eGFR, albuminuria, type 2 diabetes mellitus, sexual dysfunction, erectile dysfunction, female sexual dysfunction

## Abstract

*Background and Objectives*: Diabetic kidney disease (DKD), expressed either as albuminuria, low estimated glomerular filtration rate (eGFR) or both, and sexual dysfunction (SD), are common complications among type 2 diabetes mellitus (T2DM) patients. This study aims to assess whether an association exists between DKD and SD, erectile dysfunction (ED) or female sexual dysfunction (FSD) in a T2DM population. *Materials and Methods*: A cross-sectional study was designed and conducted among T2DM patients. The presence of SD was assessed using the International Index of Erectile Function and the Female Sexual Function Index questionnaires for males and females, respectively, and patients were evaluated for DKD. *Results*: Overall, 80 patients, 50 males and 30 females, agreed to participate. Sexual dysfunction was present in 80% of the study population. Among the participants, 45% had DKD, 38.5% had albuminuria and/or proteinuria and 24.1% had an eGFR below 60 mL/min/1.73 m^2^. The eGFR was associated with SD, ED and FSD. Moreover, SD and ED were proven as significant determinants for lower eGFR values in multiple linear regression analyses. DKD was associated with lower lubrication scores and eGFR was associated with lower desire, arousal, lubrication and total scores; however, the multivariate linear regression analyses showed no significant associations between them. Older age resulted in significantly lower arousal, lubrication, orgasm and total FSFI scores. *Conclusions*: SD is commonly encountered in older T2DM patients and DKD affects almost half of them. The eGFR has been significantly associated with SD, ED and FSD, while SD and ED were proven to be significant determinants for the eGFR levels.

## 1. Introduction

Type 2 diabetes mellitus (T2DM) affects a significant proportion of the world population, as it is estimated that 6.28% of people are affected globally, corresponding to 462 million individuals, while the prevalence is expected to rise even further in the years to come [1]. T2DM patients are at risk of developing macrovascular and microvascular complications, and these complications may even be evident at the time of the diagnosis of T2DM [2,3]. As for microvascular complications, specifically, diabetic retinopathy, nephropathy, autonomic neuropathy and peripheral neuropathy may affect from 20% to 60% of T2DM patients [4,5,6,7]. Diabetes duration and inadequate glycemic control are significant determinants of the emergence of microvascular complications [8,9].

Diabetic kidney disease (DKD) is defined as the presence of renal impairment in diabetic patients provided that other causes of renal impairment are excluded. Requirements for the diagnosis are a persistent drop in the glomerular filtration rate (GFR) below 60 mL/min/1.73 m^2^ and/or the presence of increased albuminuria ≥30 mg/g creatinine for at least three months [10,11]. DKD is commonly encountered in diabetics, as it affects 20–40% of the diabetic population [4,12,13]. Moreover, diabetes mellitus (DM) is the major cause of end-stage renal disease (ESRD) in the developed world, accounting for half of all cases [10]. DKD in type 2 diabetes mellitus (T2DM) patients does not necessarily follow the five-staged progression that was previously described for diabetic nephropathy, from hyperfiltration towards the development of albuminuria and finally ESRD, as GFR may be reduced without the coexistence of albuminuria [13,14]. Non-albuminuric DKD has been associated with the female gender and lower levels of HbA1c, while the associations for diabetic retinopathy and hypertension are weaker compared to albuminuric DKD [15]. Interestingly, in recent years, a drop in albuminuric and a rise in non-albuminuric DKD prevalence has been reported [13]. What is of great importance is the fact that albuminuria is a risk factor for the occurrence of cardiovascular diseases [16]. In the UK Prospective Diabetes Study (UKPDS), microalbuminuria, macroalbuminuria and elevated plasma creatinine or renal replacement therapy were associated with a 2.2-fold, 3.4-fold and 13.9-fold greater mortality risk, respectively [17]. Similarly, changes in eGFR towards lower values have been associated with an increased mortality risk [18].

Sexual dysfunction has been associated with T2DM in both males and females. To begin with, erectile dysfunction (ED), defined as the persistent or recurrent inability to achieve or maintain penile erection sufficient for sexual satisfaction, is estimated to affect diabetic patients 3.5 times more often with an estimated prevalence of 66.3% for T2DM in a large meta-analysis [19,20]. ED in diabetic males is difficult to treat and associated with worse quality of life [21,22]. The etiology of ED in diabetes is multifactorial, with endothelial dysfunction, hypogonadism, autonomic neuropathy and insulin resistance contributing to its emergence [23]. Finally, based on the artery-size hypothesis, ED is considered a cardiovascular disease risk factor, further enhancing the role of its early recognition and management in clinical practice [24]. 

Female sexual dysfunction (FSD) in T2DM women is often neglected and underreported, probably due to the reluctance of patients and clinicians to address the issue. FSD is estimated to affect 68.6% of diabetic women based on the results of a recent meta-analysis, whereas all the domains of female sexual function are affected, as they are expressed through the FSFI questionnaire [25]. In women, previous studies assessing the effects of diabetes on sexual function have shown unequivocal results. In particular, some have shown no connection between the presence of FSD and diabetes, while other studies have shown that there are significant associations between diabetes and disorders of female sexual function [26,27,28]. For example, in a study among young pre-menopausal women aged <45 years, the presence of diabetes resulted in lower sexual function scores for sexual drive, arousal, lubrication, orgasm and overall satisfaction compared to healthy controls [29]. Furthermore, as with older age, diabetes has been associated with low sexual desire in both males and females [30]. Finally, a systematic review and meta-analysis showed that T2DM was associated with a 2.49 risk for FSD, although when considering post-menopausal women alone, the higher prevalence of FSD in diabetic women was non-significant [31]. Unlike ED, the pathophysiologic mechanisms implicated in FSD are more complex and a clear association between FSD and cardiovascular disease has not been established [32]. 

The association among albuminuria, eGFR and ED has been explored in previous studies [33,34,35]. Similarly, other studies examined the relationship between FSD and diabetic kidney disease; however, only a few reports exist in the literature compared with ED [36,37,38,39]. This study aims to assess whether sexual dysfunction, either ED or FSD, is associated with indices of renal function in T2DM patients. 

## 2. Materials and Methods

The DIAbetic COMplications and Erectile Dysfunction study (DIACOMED) is a cross-sectional study designed and conducted under the principles of the Helsinki Declaration and approved by the Bioethics Committee of the Aristotle University of Thessaloniki (Protocol number: 1649, date of approval: 21 November 2018). All subjects gave informed consent before their enrolment in the study. The study population consisted of consecutive T2DM patients, males and females, who visited the outpatient clinic of the Second Propedeutic Department of Internal Medicine of the Aristotle University of Thessaloniki and agreed to participate from November 2018 until November 2020. A prior diagnosis of T2DM was required for the enrolment, while those aged below 18 years, with an inability or unwillingness to participate, with a history of alcohol or drug abuse or with an acute illness were excluded from the study. 

A medical history and vital signs (blood pressure, pulse rate, weight, height) were collected and recorded. A medication history was also obtained. Blood pressure was measured in the office using an automated oscillometric device in the sitting position, while ambulatory blood pressure monitoring (ABPM) over 24 h was also performed, all following the European Society of Hypertension guidelines [40]. Blood samples were collected after an 8 h fasting period for the evaluation of fasting plasma glucose (FPG), glycated hemoglobin (HbA1c), liver function tests, lipids [total cholesterol (TChol), triglycerides (TG), high-density lipoprotein cholesterol (HDL-c), low-density lipoprotein cholesterol (LDL-c)] and renal function tests (plasma urea and creatinine). A 24 h urine collection was performed in all patients, and samples were assessed for the presence of proteinuria and/or albuminuria.

The Chronic Kidney Disease Epidemiology Collaboration (CKD-EPI) equation was used for the calculation of the estimated glomerular filtration rate (eGFR) [41]. Renal function was subdivided into 5 categories, based on the eGFR: G1 for an eGFR ≥ 90 mL/min/1.73 m^2^, G2 for an eGFR 60–89 mL/min/1.73 m^2^, G3 for an eGFR 30–59 mL/min/1.73 m^2^, G4 for an eGFR 15–29 mL/min/1.73 m^2^ and finally G5 for an eGFR below 15 mL/min/1.73 m^2^ [11]. Apart from that, patients were divided into two groups: those with an eGFR above 60 mL/min/1.73 m^2^ and those with values below that level.

Moreover, based on the 24 h urine collection results, albuminuria was divided into three categories: A1 (normal to mildly increased albuminuria), A2 (moderately increased albuminuria) and A3 (severely increased albuminuria), as stated by the 2012 KDIGO guidelines [11]. An albumin excretion rate (AER) and a protein excretion rate (PER) below 30 mg/24 h and 150 mg/24 h, respectively, were indicative of A1 albuminuria, an AER and PER 30–300 mg/24 and 150–500 mg/24 h, respectively, were indicative of A2 albuminuria and, finally, an AER above 300 mg/24 h and a PER above 500 mg/24 h were indicative of A3 albuminuria. An eGFR below 60 mL/min and/or A2 and A3 albuminuria stages were required for the diagnosis of DKD [10].

Sexual function was assessed using the International Index of Erectile Function (IIEF) questionnaire in males and the Female Sexual Function Index (FSFI) questionnaire in females. In detail, the IIEF questionnaire consists of 15 items that evaluate erectile function (EF), orgasmic function, sexual desire, intercourse satisfaction and overall satisfaction in males. A score below 25 in erectile function is indicative of erectile dysfunction (ED), while patients with ED are further classified into four categories: those with mild ED (IIEF-EF scores 22–25), those with mild to moderate ED (IIEF-EF scores 17–21), those with moderate ED (IIEF-EF scores 11–16) and finally those with severe ED (IIEF-EF scores 6–10) [42]. Similarly, the FSFI questionnaire consists of 19 items and examines the six domains of female sexual function: desire, arousal, lubrication, orgasm, satisfaction and pain. A total score below 26.55 is indicative of female sexual dysfunction (FSD) [43,44].

The Statistical Package for Social Sciences (IBM SPSS Statistics, 28.0 version) was used for statistical analyses. All analyses were performed at the 0.05 level of significance. For qualitative variables, the χ^2^ test was used, while for quantitative variables the Kolmogorov–Smirnov test and the Shapiro–Wilk test were used, based on the number of variables. Variables with a normal distribution were expressed as mean ± SD and those with an abnormal distribution as the median (25–75% percentiles). Student’s *t*-test, ANOVA and Pearson’s correlation analysis were used for those variables with a normal distribution, while Mann–Whitney, Kruskal–Wallis and Spearman’s correlation analysis were used for variables with an abnormal distribution. For ED and FSD, univariate and multivariate logistic regression analyses were performed, based on the study findings or other published data, while univariate and multiple linear logistic regression analyses were also implemented for the different domains of the IIEF and FSFI questionnaires separately. 

## 3. Results

From November 2018 to November 2020, 80 patients, 50 males and 30 females, agreed to participate in the study. The median age of the participants was 65 (56–71) years, and the median diabetes duration was 10 (6–15.75) years, while glycemic control was far from ideal in most participants, as 61.3% had an HbA1c above 7% and the median HbA1 was 7.15 (6.4–8.2)%. Regarding comorbidities, 78.8% had hypertension, 35% had a history of cardiovascular disease (coronary artery disease, stroke or peripheral arterial disease) and 75.3% had dyslipidemia. As for hypoglycemic medication, 85.1% received metformin, 21.6% a sodium-glucose cotransporter-2 inhibitor (SGLT2i), 16.2% a glucagon-like peptide-1 receptor agonist (GLP1-RA), 24.3% a dipeptidyl peptidase-4 inhibitor (DPP-4i), 29.7% were on insulin treatment and finally, 6.8% received sulfonylureas. Moreover, 60.8% of the study population were on treatment with a renin-angiotensin-aldosterone system inhibitor, 37.8% of them received a calcium channel blocker, 23% a thiazide diuretic, 39.2% a beta blocker, 9.5% a loop diuretic, 6.8% a mineralocorticoid receptor antagonist and 55.4% a statin. No statistically significant differences were observed among those with or without sexual dysfunction, ED or FSD, respectively, apart from the significantly higher use of statins among women with FSD, as depicted in Supplementary Tables S1–S3.

Overall, 80% had sexual dysfunction: 86% of male participants had ED and 73.3% of female participants had FSD. Among them, 10% were single, 2.5% were in a relationship, 72.5% were married, 3.8% were divorced and 11.3% were widowed. The median erectile function score was 18 (9–22) and the median FSFI total score was 19.1 (10.3–25.92), while further details regarding the median scores of the different domains of the IIEF and FSFI questionnaires are shown in Table 1 and Table 2, respectively. 

Among those with ED, 23.2% had mild ED, 32.5% had mild to moderate ED, 11.6% had moderate ED and finally, 32.5% had severe ED. Those with sexual dysfunction were significantly older than those without (median age 65.5 vs. 52 years, U = 698.5, *p* = 0.025) and had lower levels of HbA1c (median HbA1c 6.45% vs. 8.65%, U = 329, *p* = 0.028), as depicted in Table 3. However, age and HbA1c values did not differ significantly among those with or without ED or FSD, respectively. 

Diabetic kidney disease was present in 45% of the population. Specifically, 38.5% had albuminuria and/or proteinuria above 30 mg/24 h and 150 mg/24 h, respectively (24.4% with A2, and 14.1% with A3 albuminuria stage), while 24.1% had an eGFR below 60 mL/min/1.73 m^2^. Based on the eGFR values, 25.7%, 54.1%, 17.6% and 2.7% had G1, G2, G3 and G5 stages of renal function, respectively. As for other diabetic complications, 23.8% had retinopathy, 23.8% had diabetic peripheral neuropathy and 44.9% had cardiac autonomic neuropathy. The prevalence of DKD did not differ among those with and without SD, ED or FSD (Table 3). Similarly, no differences were found among the aforementioned study groups and the prevalence of albuminuria. On the contrary, the presence of SD, ED and FSD was significantly associated with lower eGFR values (for SD: mean eGFR 71.77 (19.72) with vs. 96 (6.48) without SD, t (77) = 3.321, *p* = 0.001; for ED: mean eGFR 76.57 (20.58) with vs. 97 (8.48) without ED, t (47) = 2.446, *p* = 0.018; for FSD: mean eGFR 66.36 (15.19) with vs. 81.5 (13.98) without FSD, t (28) = 2.46, *p* = 0.02). Moreover, the presence of SD was associated with an eGFR below 60 mL/min/1.73 m^2^ [x^2^ (1, N = 79) = 6.353, *p* = 0.012]. 

No association was found among the different aspects of male sexual function, as they are expressed by the IIEF questionnaire, and the presence of DKD, albuminuria or lower eGFR values. As for the individual components of female sexual function, the presence of DKD was significantly associated with lower lubrication scores (median lubrication score 1.35 vs. 4.05, U = 61.5, *p* = 0.048), while an eGFR below 60 mL/min/1.73 m^2^ was associated with lower arousal and lubrication scores (for arousal: median score 1.2 vs. 3.3, U = 40, *p* = 0.048; for lubrication: median score 1.2 vs. 3.9, U = 29.5, *p* = 0.01). Likewise, the eGFR was significantly positively correlated with desire, arousal, lubrication and total FSFI scores, as depicted in Table 4. Among other factors, age was significantly associated with the presence of SD (median age 59 vs. 66 years for those without and with SD, respectively, U = 698.5, *p* = 0.025), and it was also negatively correlated with eGFR and with the total FSFI score, as well as with the FSFI domains of desire, arousal, lubrication, orgasm and pain (Supplementary Table S4). 

The univariate linear regression analyses showed that age, eGFR and HbA1c were associated with the FSFI desire, pain, arousal and total scores, and age and eGFR alone were associated with the FSFI lubrication and orgasm scores. However, the multivariate regression analyses revealed that among age, eGFR and HbA1c, only age remained a significant determinant for the FSFI domains of arousal, lubrication and orgasm, as well as for the FSFI total score (R^2^ = 0.414, F(3, 26) = 6.118, *p* = 0,003 for arousal; R^2^ = 0.542, F(3, 26) = 14.92, *p* = 0.001 for lubrication; R^2^ = 0.407, F(3, 26) = 5.941, *p* = 0.003 for orgasm; and R^2^ = 0.516, F(3, 26) = 9.244, *p* = 0.001 for FSFI total scores, respectively). Specifically, for every increase in age by ten years, a drop by 0.7 (β = −0.074, *p* = 0.032), 1.2 (β = −0.121, *p* = 0.001), 1 (β = −0.103, *p* = 0.005) and 4.5 (β = −0.456, *p* = 0.006) points in the FSFI arousal, lubrication, orgasm and total scores, respectively, was expected. Regarding the eGFR, the univariate linear regression analyses showed that it was associated with age, diabetes duration and the presence of cardiovascular disease, SD, ED or FSD. On the contrary, the multivariate linear regression analyses revealed that, among other variables, only SD and ED remained significant determinants of the eGFR levels (R^2^ = 0.277, F(4, 74) = 7.098, *p* = 0.001 and R^2^ = 0.340, F(4, 44) = 5.672, *p* = 0.001, respectively), while FSD was not (R^2^ = 0.434, F(3, 26) = 6.646, *p* = 0.002) (Table 5). 

## 4. Discussion

In this cross-sectional study involving relatively old T2DM patients, sexual dysfunction, either ED or FSD, was present in 80% of the overall study population. Among them, 45% had DKD, 38.5% had albuminuria and/or proteinuria and 24.1% had an eGFR below 60 mL/min/1.73m^2^. The eGFR was associated with SD, ED and FSD, whereas DKD and albuminuria were not. Moreover, SD and ED were proven as significant determinants for lower eGFR values in the multiple linear regression analyses. As for the specific domains of female sexual function, DKD was associated with lower lubrication scores and eGFR was associated with lower desire, arousal, lubrication and total scores; however, after the multivariate linear regression analyses were performed, no significant associations remained among them. On the contrary, age was proven to contribute significantly to lower arousal, lubrication, orgasm and total FSFI scores. 

In DM, albuminuria is caused by many factors, most of which originate from inflammatory processes [16,45]. Albumin glycation, reactive oxygen species (ROS) formation, advanced glycation end products (AGEs) accumulation and other toxins result in vascular damage and the subsequent development of albuminuria, while in the emergence of inflammation, hyperinsulinemia seems to participate as well [16,46]. Meanwhile, increased sodium and protein intake and poor blood pressure control all seem to contribute [47,48,49]. On the contrary, measures to optimize glycemic control result in lower rates of DKD [50]. Similar mechanisms of oxidative stress and endothelial dysfunction are involved in the emergence of ED in T2DM patients [51]. However, in our study, erectile dysfunction was not associated with albuminuria. This finding contradicts the results from previous studies, where erectile dysfunction was associated with the presence of albuminuria in T2DM patients. Specifically, a cross-sectional study conducted in Japan revealed that diabetics with macroalbuminuria were more likely to have low IIEF scores than those with normoalbuminuria [34]. Similarly, another study among T2DM Egyptian patients revealed that a higher urinary albumin to creatinine ratio (UACR) was a significant predictor for a diagnosis of ED [35]. In another study among Chinese T2DM patients, ED was diagnosed with the use of the IIEF-5 score in 84.3% of the patients. Among them, 58.3% had mild-to-moderate ED and 41.7% had severe ED. ED severity expressed by lower IIEF-5 scores was associated with higher UACR values and lower eGFR values, while after the multivariable logistic regression analysis, albuminuria remained an independent predictor for ED [52]. The observed difference between our study findings and previous studies might be attributed to differences in the study population, such as age, comorbidities, race and geographic variation. 

In the past years, a shift in the phenotype of DKD has been observed, with an increasingly higher prevalence of non-albuminuric kidney disease. About half of DM patients with an eGFR below 60 mL/min/m^2^ have normal albumin excretion [15]. The reasons for this trend are not clear; however, it is hypothesized that the wider prescription of renin-angiotensin inhibitors in clinical practice and the better pharmacologic control of hypertension and dyslipidemia may have contributed to it. Moreover, since non-albuminuric kidney disease is not associated with glycemic control and other microvascular complications to the same extent as albuminuric kidney disease, it is suggested that the underlying pathophysiologic mechanism is macroangiopathy rather that microangiopathy [53,54]. Another possible explanation is that the reduced eGFR is a consequence of repeated or unresolved episodes of acute renal failure that may result in progressive glomerulosclerosis and tubulointerstitial fibrosis [54,55]. Similar to our study, low eGFR has been associated with erectile dysfunction in previous studies. In particular, a cross-sectional study among T2DM patients conducted in a Chinese population found that 82% of the participants reported having ED. Age and diabetes duration were significant predictors of the development of ED. Neuropathy, albuminuria, higher UACR and serum creatinine values and lower eGFR values were observed in the cohort of ED patients compared to those without ED. When adjusted for age and diabetes duration, albuminuria and lower eGFR remained significantly correlated with ED. Specifically, the OR of ED was 2.48 and 4.49 for microalbuminuria and macroalbuminuria, respectively. The OR of severe ED, however, was 2.87 for microalbuminuria and 10.21 for macroalbuminuria, larger than those for ED, and those with severe ED had lower eGFR values compared to those with no ED [33]. 

Diabetes affects many aspects of female sexual function. Hyperglycemia may lead to higher rates of infection and reduced lubrication, while neuropathy, endothelial dysfunction and atherosclerotic damage seem to contribute to the emergence of FSD as well [23]. However, only a few studies have assessed its association with diabetic kidney disease. A cross-sectional study among pre-menopausal diabetic women showed that albuminuria and diabetic nephropathy were significantly associated with the presence of FSD [38]. Similarly, another cross-sectional study among middle-aged women found that albuminuria was significantly correlated with sexual dysfunction [39]. Moreover, among diabetic Jordanian women, the presence of complications such as nephropathy and retinopathy was significantly associated with the presence of FSD [36]. On the contrary, another study among Chinese T2DM women and healthy controls found no association between FSD and diabetic nephropathy, whereas age and diabetic neuropathy were proven as significant determinants for worse sexual function [37]. 

Our study has several limitations. As it is a cross-sectional study, causality cannot be established. Furthermore, our sample size was small and with many comorbidities, which may have influenced the results. Furthermore, among those with sexual dysfunction and those without, differences were observed regarding diabetes duration and glycemic control. In particular, those with SD had a shorter diabetes duration and better glycemic control compared to those without SD. As both factors are associated with the emergence of diabetic complications, such as diabetic nephropathy, this observed difference could result in the elimination of any probable correlation between albuminuria and SD. Likewise, 62.5% of the study population were on treatment with an angiotensin-converting enzyme inhibitor or an angiotensin receptor blocker, agents that are well known for their positive effect on albuminuria progression, thus further influencing the results. Finally, the diagnosis of diabetic nephropathy was based on one assessment and was not verified in follow-up measurements, which are required according to the definition of diabetic nephropathy.

## 5. Conclusions

To conclude, sexual dysfunction is commonly encountered in older T2DM patients, either males or females, and DKD affects almost half of them. Among DKD, albuminuria and eGFR, the latter has been significantly associated with SD, ED and FSD, while SD and ED were proven to be significant determinants for the eGFR levels. However, further, larger, prospective randomized controlled studies are needed to verify whether these associations exist and to further ascertain if measures to control diabetic kidney disease can have an impact on the sexual function of T2DM patients. 

## Figures and Tables

**Table 1:** IIEF scores among patients with and without DKD.

	Total Patients *n* = 50	With DKD *n* = 24	Without DKD *n* = 26	*p*
Erectile function	18 (9–22)	20 (11–22.5)	18 (9–22)	0.640
Orgasmic function	8 (5–9)	8 (5–9)	7.5 (5–9)	0.695
Sexual desire	7.5 (4–9)	7.5 (4–9)	7.5 (5–9)	0.768
Intercourse satisfaction	9 (6–11)	10 (5–11)	9 (6–11)	0.906
Overall satisfaction	6 (4–8)	6.5 (4.5–8)	5 (4–7)	0.243

Expressed as median (25th–75th percentile). DKD: diabetic kidney disease; IIEF: International Index of Erectile Function Score.

**Table 2:** FSFI scores among patients with and without DKD.

	Total Patients *n* = 30	With DKD *n* = 12	Without DKD *n* = 18	*p*
Desire	2.4 (1.2–3.6)	1.2 (1.2–3)	2.7 (1.2–3.6)	0.465
Arousal	2.7 (1.2–3.67)	1.2 (1.2–3.6)	3.15 (1.5–3.6)	0.232
Lubrication	3.6 (1.2–4.65)	1.35 (1.2–3.75)	4.05 (2.4–5.1)	0.048
Orgasm	3.6 (1.2–4.4)	1.8 (1.2–4.4)	3.8 (2.8–4.4)	0.391
Satisfaction	4 (3.6–5.6)	3.8 (3.6–4.4)	4.2 (3.6–5.6)	0.518
Pain	3 (1.6–5.2)	2.2 (1.2–4.4)	3.2 (2–5.6)	0.146
Total score	19.1 (10.3–25.92)	12 (9.6–21.25)	21.85 (15.9–26)	0.158

Expressed as median (25th–75th percentile). DKD: diabetic kidney disease; FSFI: Female Sexual Function Index.

**Table 3:** Main demographic and clinical characteristics of the study population according to the presence of SD, ED and FSD.

	With SD *n* = 64	Without SD *n* = 16	*p*	With ED *n* = 43	Without ED *n* = 7	*p*	With FSD *n* = 22	Without FSD *n* = 8	*p*
Age (y) **	65.5 (60–73)	52 (48–59)	0.025	64 (56–72)	59 (53–65)	0.41	65.5 (61–73)	59 (51–65.5)	0.05
Diabetes									
Diabetes duration (y) **	10 (6–15)	14 (7.5–21)	0.646	8 (5–10)	17 (10–24)	0.394	14.5 (7–20)	13.5 (6.5–20.5)	1
HbA1c (%) **	6.45 (6.3–7.4)	8.65 (8.3–9.95)	0.028	7.25 (6.4–8)	9.95 (8.7–11.2)	0.157	6.75 (6.3–7.7)	7.85 (6.85–8.7)	0.078
HbA1c below 7%	27 (42.2%)	4 (25%)	0.207	15 (34.9%)	1 (14.3%)	0.279	12 (54.5%)	3 (37.5%)	0.409
FPG (mg/dl) **	132.5 (113–172)	190.5 (134–218)	0.963	172 (139–194)	161.5 (84–239)	0.493	127 (113–163)	144 (124–154)	0.304
DKD									
DKD	30 (46.9%)	6 (37.5%)	0.50	20 (46.5%)	4 (57.1%)	0.602	10 (45.5%)	2 (25%)	0.312
Albuminuria	23 (37.7%)	6 (37.5%)	0.988	16 (40%)	4 (57.1%)	0.397	7 (31.8%)	2 (25%)	0.719
Stage A1	38 (61.3%)	10 (62.5%)	0.528	24 (58.5%)	3 (42.9%)	0.498	15 (68.2%)	6 (75%)	0.677
Stage A2	14 (22.6%)	5 (31.3%)	0.528	9 (22%)	3 (42.9%)	0.498	5 (22.7%)	2 (25%)	0.677
Stage A3	10 (16.1%)	1 (6.3%)	0.528	8 (19.5%)	1 (14.3%)	0.498	2 (9.1%)	0 (0%)	0.677
eGFR *	71.77 (19.72)	96 (6.48)	0.001	76.57 (20.58)	97 (8.48)	0.018	66.36 (15.19)	81.5 (13.98)	0.02
eGFR below 60 mL/min/1.73 m^2^	19 (30.2%)	0 (0%)	0.012	12 (28.6%)	0 (0%)	0.104	7 (31.8%)	0 (0%)	0.068
Comorbidities									
Dyslipidemia	48 (77.4%)	10 (66.7%)	0.386	31 (75.6%)	4 (57.1%)	0.31	18 (81.8%)	5 (71.4%)	0.554
Dyslipidemia duration **	10 (5–14)	5.5 (1–10)	0.462	7 (3–10)	10 (10–10)	0.132	10.5 (7.5–14.5)	1 (1–1)	0.044
Hypertension	52 (81.3%)	11 (68.8%)	0.274	35 (81.4%)	4 (57.1%)	0.151	18 (81.8%)	6 (75%)	0.68
Hypertension duration *	9.64 (7.29)	8.5 (7.32)	0.483	6.78 (4.12)	5.5 (6.36)	0.492	11.5 (7.95)	16.25 (1.5)	0.128
CVD disease (stroke, PAD, CAD)	24 (37.5%)	4 (25%)	0.348	20 (46.5%)	3 (42.9%)	0.857	5 (22.7%)	0 (0%)	0.14

Expressed as *n*(%), mean (SD) *, median (25th–75th percentile) **. CAD: coronary artery disease; CVD: cardiovascular disease; DKD: diabetic kidney disease; ED: erectile dysfunction; FPG: fasting plasma glucose; FSD: female sexual dysfunction; eGFR: estimated glomerular filtration rate; HbA1c: glycated hemoglobin; PAD: peripheral artery disease; SD: sexual dysfunction.

**Table 4:** Correlations among FSFI domains and eGFR in female patients.

	*r*	*p*
Desire	0.373	0.043
Arousal	0.436	0.016
Lubrication	0.475	0.008
Orgasm	0.324	0.08
Satisfaction	0.111	0.558
Pain	0.361	0.05
Total score	0.362	0.049

eGFR: estimated glomerular filtration rate; FSFI: Female Sexual Function Index.

**Table 5:** Multiple linear regression results for eGFR. (a). The associations among SD, age, diabetes duration and cardiovascular disease and eGFR. (b). The associations among age, diabetes duration, cardiovascular disease and ED and eGFR. (c). The associations among age, diabetes duration and FSD and eGFR.

Model	Unstandardized Coefficients		Standardized Coefficients	t	Sig.	95.0% CI for B	
	B	Std. Error	Beta			Lower Bound	Upper Bound
(a)							
(Constant)	124.502	13.912		8.950	0.001	96.782	152.221
Age	−0.489	0.239	−0.223	−2.041	0.045	−0.966	−0.012
Diabetes duration	−0.580	0.307	−0.198	−1.890	0.063	−1.191	0.031
CVD disease	−8.251	4.726	−0.174	−1.746	0.085	−17.667	1.164
SD	−15.860	5.829	−0.281	−2.721	0.008	−27.475	−4.245
(b)							
(Constant)	124.019	19.657		6.309	0.000	84.403	163.635
Age	−0.214	0.334	−0.089	−0.642	0.524	−0.888	0.459
Diabetes duration	−0.923	0.431	−0.291	−2.144	0.038	−1.791	−0.056
CVD disease	−16.315	6.421	−0.314	−2.541	0.015	−29.255	−3.376
ED	−24.487	9.262	−0.331	−2.644	0.011	−43.152	−5.821
(c)							
(Constant)	135.405	16.446		8.233	0.000	101.601	169.210
Age	−0.905	0.268	−0.543	−3.372	0.002	−1.457	−0.353
Diabetes duration	−0.121	0.347	−0.052	−0.349	0.730	−0.835	0.592
FSD	−7.452	5.764	−0.208	−1.293	0.207	−19.301	4.397

(a) CI: confidence interval; CVD: cardiovascular disease; eGFR: estimated glomerular filtration rate; SD: sexual dysfunction. (b) CI: confidence interval; CVD: cardiovascular disease; ED: erectile dysfunction; eGFR: estimated glomerular filtration rate. (c) CI: confidence interval; eGFR: estimated glomerular filtration rate; FSD: female sexual dysfunction.

## Data Availability

Data may be available on request due to privacy restrictions.

## Supplementary Materials

The following supporting information can be downloaded at: https://www.mdpi.com/article/10.3390/medicina59050969/s1, Table S1: Medication of the study population according to the presence of sexual dysfunction; Table S2: Medication of the study population according to the presence of erectile dysfunction; Table S3: Medication of the female study population based on female sexual function; Table S4: Correlations between age and FSFI domains and eGFR in female patients.
