# Supplementary material for: The Associations between Kidney Function and Sexual Dysfunction among Males and Females with Type 2 Diabetes Mellitus
Source: Medicina (Kaunas). 2023 May 17;59(5):969. doi: 10.3390/medicina59050969 (PMC10220764; doi:10.3390/medicina59050969)
Supplement: Supplementary file 1 [file medicina-59-00969-s001.zip › Supplementary table 1.pdf]

Supplementary Table S1. Medication of the study population according to the presence of sexual dysfunction.

	Total patients N=80	Without sexual dysfunction N=16	With sexual dysfunction N=64	P
Metformin	67 (84.8%)	14 (87.5%)	53 (84.1%)	0.737
GLP1-RA	12 (15%)	2 (12.5%)	10 (15.6%)	0.754
SGLT2i	18 (22.5%)	3 (18.8%)	15 (23.4%)	0.688
Glitazones	1 (1.3%)	0 (0%)	1 (1.6%)	0.615
Sulfonylurias	7 (8.8%)	1 (6.3%)	6 (9.4%)	0.692
DPP-4 inhibitors	23 (28.7%)	4 (25%)	19 (29.7%)	0.711
Insulin	23 (28.7%)	5 (31.3%)	18 (28.1%)	0.805
ACEi/ARB	50 (62.5%)	9 (56.3%)	41 (64.1%)	0.564
CCB	29 (36.3%)	7 (43.8%)	22 (34.4%)	0.485
Thiazide diuretics	18 (22.5%)	2 (12.5%)	16 (25%)	0.284
Loop diuretics	9 (11.3%)	0 (0%)	9 (14.1%)	0.111
Beta blocker	31 (38.8%)	5 (31.3%)	26 (40.6%)	0.491
MRA	6 (7.5%)	0 (0%)	6 (9.4%)	0.203
Statins	46 (57.5%)	6 (37.5%)	40 (80%)	0.07

Expressed as n(%)

ACEi: Angiotensin-converting enzyme inhibitors; ARB: Angiotensin receptor blockers; CCB: Calcium channel blocker; DPP-4: Dipeptidyl peptidase-4; GLP1-RA: Glucagon-like peptide-1 receptor agonists; MRA: Mineralocorticoid receptor antagonists; SGLT2i: Sodium-glucose Cotransporter-2 inhibitors
