# Supplementary material for: The Associations between Kidney Function and Sexual Dysfunction among Males and Females with Type 2 Diabetes Mellitus
Source: Medicina (Kaunas). 2023 May 17;59(5):969. doi: 10.3390/medicina59050969 (PMC10220764; doi:10.3390/medicina59050969)
Supplement: Supplementary file 1 [file medicina-59-00969-s001.zip › Supplementary table 2.pdf]

Supplementary Table S2. Medication of the male study population according to the presence of erectile function.

	Without ED<br>N=7	With ED<br>N=43	P
Metformin	7 (100%)	35 (81.4%)	0.213
GLP1-RA	1 (14.3%)	5 (11.6%)	0.841
SGLT2i	2 (28.6%)	8 (18.6%)	0.541
Glitazones	0 (0%)	0 (0%)	-
Sulfonylurias	1 (14.3%)	4 (9.3%)	0.684
DPP-4 inhibitors	2 (28.6%)	13 (30.2%)	0.929
Insulin	3 (42.9%)	11 (25.6%)	0.345
ACEi/ARB	3 (42.9%)	26 (60.5%)	0.381
CCB	3 (42.9%)	15 (34.9%)	0.684
Thiazide diuretics	0 (0%)	11 (25.6%)	0.130
Loop diuretics	0 (0%)	9 (20.9%)	0.181
Beta blocker	3 (42.9%)	20 (46.5%)	0.857
MRA	0 (0%)	4 (9.3%)	0.40
Statins	4 (57.1%)	24 (55.8%)	0.948

Expressed as n(%)

ACEi: Angiotensin-converting enzyme inhibitors; ARB: Angiotensin receptor blockers; CCB: Calcium channel blocker; DPP-4: Dipeptidyl peptidase-4; ED: erectile dysfunction; GLP1-RA: Glucagon-like peptide-1 receptor agonists; MRA: Mineralocorticoid receptor antagonists; SGLT2i: Sodium-glucose Cotransporter-2 inhibitors
