# Supplementary material for: The Associations between Kidney Function and Sexual Dysfunction among Males and Females with Type 2 Diabetes Mellitus
Source: Medicina (Kaunas). 2023 May 17;59(5):969. doi: 10.3390/medicina59050969 (PMC10220764; doi:10.3390/medicina59050969)
Supplement: Supplementary file 1 [file medicina-59-00969-s001.zip › Supplementary table 3.pdf]

Supplementary Table S3. Medication of the female study population based on female sexual function.

	Without FSD N=8	With FSD N=22	P
Metformin	7 (87.5%)	18 (85.7%)	0.901
GLP1-RA	1 (12.5%)	5 (22.7%)	0.536
SGLT2i	1 (12.5%)	7 (31.8%)	0.290
Glitazones	0 (0%)	1 (4.5%)	0.540
Sulfonylurias	0 (0%)	2 (9.1%)	0.377
DPP-4 inhibitors	2 (25%)	6 (27.3%)	0.901
Insulin	2 (25%)	7 (31.8%)	0.719
ACEi/ARB	5 (62.5%)	16 (72.7%)	0.589
CCB	4 (50%)	7 (31.8%)	0.361
Thiazide diuretics	2 (25%)	5 (22.7%)	0.896
Loop diuretics	0 (0%)	0 (0%)	-
Beta blocker	2 (25%)	6 (27.3%)	0.901
MRA	0 (0%)	2 (9.1%)	0.377
Statins	2 (25%)	16 (72.7%)	0.018

Expressed as n(%)

ACEi: Angiotensin-converting enzyme inhibitors; ARB: Angiotensin receptor blockers; CCB: Calcium channel blocker; DPP-4: Dipeptidyl peptidase-4; FSD: female sexual function; GLP1-RA: Glucagon-like peptide-1 receptor agonists; MRA: Mineralocorticoid receptor antagonists; SGLT2i: Sodium-glucose Cotransporter-2 inhibitors
