# Supplementary material for: The Associations between Kidney Function and Sexual Dysfunction among Males and Females with Type 2 Diabetes Mellitus
Source: Medicina (Kaunas). 2023 May 17;59(5):969. doi: 10.3390/medicina59050969 (PMC10220764; doi:10.3390/medicina59050969)
Supplement: Supplementary file 1 [file medicina-59-00969-s001.zip › Supplementary table 4.pdf]

Supplementary Table S4. Correlations among age and FSFI domains and eGFR in female patients

	r	P
Desire	-0.487	0.006
Arousal	-0.515	0.004
Lubrication	-0.742	0.001
Orgasm	-0.547	0.002
Satisfaction	-0.258	0.169
Pain	-0.508	0.004
Total score	-0.610	0.001
eGFR	-0.488	0.001

eGFR: estimated glomerular filtration rate; FSFI: Female Sexual Function Index
